# Increasing handgrip strength via post-hypnotic suggestions with lasting effects

**DOI:** 10.1038/s41598-024-73117-0

**Published:** 2024-10-14

**Authors:** Ulrike Nieft, Marleen Schlütz, Barbara Schmidt

**Affiliations:** 1 Institute for Biology, University of Leipzig, Leipzig, Germany; 2 Institute for Psychology, Friedrich Schiller University Jena, Jena, Germany; 3 Institute for Psychosocial Medicine, Psychotherapy and Psychooncology, Jena University Hospital, Stoystraße 3, 07743 Jena, Germany

**Keywords:** Hypnosis, Post-hypnotic suggestion, Handgrip strength, Sports performance, Rehabilitation, Long-term effects, Motor control, Neurophysiology, Human behaviour, Neuroscience, Physiology, Psychology, Neurology

## Abstract

In our study, we suggested participants to feel strong during hypnosis and tested if that affected their handgrip strength. Handgrip strength is measured via a hand dynamometer and indicates muscle strength and participants’ general medical condition. In addition, we obtained subjective ratings of strength via a visual analogue scale. We developed a hypnosis intervention to increase strength and tied the feeling of strength to a post-hypnotic power anchor. Participants could activate this power anchor afterwards to feel strong again. We also had a randomized control group that read Arnold Schwarzenegger’s autobiography instead of hypnosis. We tested the effect of the post-hypnotic power anchor on two experimental sessions separated by one week. Our data show that participants in the hypnosis group felt significantly stronger when they activated their post-hypnotic power anchor compared to their own baseline, both in the first and second experimental session. In addition, participants in the hypnosis group showed a significant increase in objective handgrip strength compared to their own baseline one week after the hypnosis session. We conclude that our hypnosis intervention primarily improved strength perception and secondary objective handgrip strength. Our intervention can help patients to improve their medical condition and athletes to improve their sport performance.

**Supplementary Information:**

The online version contains supplementary material available at 10.1038/s41598-024-73117-0.

## Introduction

From athletes seeking a competitive advantage to patients yearning for speedy recovery—using the power of mind to improve physical abilities holds immense promise. Hypnotic interventions, proven effective and used in clinical^1–5^, psychotherapeutic^6^, and sports contexts^7^, emerge as an appropriate tool for this purpose. Previous studies have shown that hypnosis can improve sports performance, for example soccer performance^8^. Participants who received hypnosis were significantly better at shooting a soccer ball at a distant target than the control group who watched soccer videos resulting in a shot or goal^8^. Schreiber^9^ demonstrated similar effects in basketball. Players who received repeated hypnosis sessions showed increased scoring during the playing season compared to the control group^9^. A recent study from our lab revealed that professional downhill mountain bikers were able to optimize their mental and physiological preparation for an upcoming race with one hypnosis session^10^. The hypnosis intervention significantly reduced anxiety and increased self-confidence. To assess physiological race preparation, we measured heart rate variability. After the hypnosis session, heart rate variability was significantly higher. High heart rate variability indicates good health and adaptability; low heart rate variability indicates stress, anxiety or health issues. In addition, athletes in the hypnosis group showed generally better race performance than athletes in the control group^10^. These hypnosis studies indicate that hypnosis affects physiological parameters. They also show that mental processes are linked to physical performance: Hypnosis can reduce pain^11^ and anxiety^12,13^ which in turn affects physiology, for example respiration rate or heart rate^14^.

Our current study investigates if hypnosis can affect handgrip strength. We chose handgrip strength because it has proven as an easy and recognized measure in clinical practice for health screening in several cases, e.g. the general medical condition^15^ or cardiovascular mortality^16^. The aim of this study is to determine whether a suggestion of being strong given during hypnosis leads to an increase in handgrip strength. We tied the feeling of being strong to a post-hypnotic power anchor that participants could activate outside of hypnosis. In previous studies, we showed that post-hypnotic suggestions work even weeks after their installation during hypnosis^17,18^. To investigate if participants could really re-activate their feeling of strength, we measured the impact of the post-hypnotic suggestion immediately after the hypnosis session and one week later using subjective and objective strength parameters.

Objective parameters have long been studied concerning the evaluation of health or sports performance. Today, we know that the mental state is crucial and strongly influences people’s performance^19^ and health^20^. Most previous studies on stress have described differences between subjective and objective parameters^21^, e.g. no significant correlations were found between cortisol levels and the perception of stress^18^. Campbell and Ehlert^21^ suggest that these findings might be due to methodological approaches or to psychophysiological differences within subjects. Because of these findings, we measure both objective and subjective strength parameters.

Our hypnosis intervention to evoke the feeling of strength is based on suggestions that have proven as useful in previous studies^22^. We aimed at increasing motivation and encouraged participants to show their full potential. We had two main predictions. The first prediction was that our hypnosis intervention increases both subjective and objective handgrip strength. The second prediction was that the post-hypnotic power anchor worked both immediately after the hypnosis session and one week later.

## Materials and methods

### Participants

We performed an a priori power analysis. Based on the effect size of post-hypnotic suggestions on subjective ratings in previous studies conducted in our laboratory^17,18,23^, an effect of d = 0.7 (Cohen’s d) was assumed for the effect of post-hypnotic strength suggestions on subjective strength. A power analysis with the program G*Power^24^ showed that a sample size of 24 participants per group is required to measure effects of this size. The alpha error probability was set to 0.05 and the test power to 0.95. Based on this power analysis, we collected data from 24 participants in the hypnosis group and 24 participants in the control group.

Participants were recruited via posters (10.5281/zenodo.10866281) and extended acquaintances. They were recruited in the cities Leipzig and Jena. We tested 24 participants in Leipzig and 24 participants in Jena with the same number of participants for the control and hypnosis groups. For better comparability, the ratio of the sexes male/female was tried to be as balanced as possible, although no specific hypothesis regarding sex was assumed. Participants were randomized to either the hypnosis or control group with an automized tool (paRANDies tool of the University Hospital Jena, *cf. Randomization & Stratification*). All recorded data were stored and processed pseudonymously.

Participants were informed about the study prior to the trial (10.5281/zenodo.10866281) and could only participate after signing the informed consent form (10.5281/zenodo.10866281). Participants in the control group received the hypnosis intervention as an audio file after the end of the study.

### Study design

The long-term effects were tested one week later on day 8 (several days later were also fine). Table 1 shows the study design with the measurement points. Baseline measurements were done on day 1, before the hypnosis intervention and on day 8, before activating the power anchor to compare the effects within participants.

**Table 1:** Study design with four measurement points (T1–T4).

Group	Day 1			Day 8	
Hypnosis	T1 Baseline	Hypnosis	T2 post-hypnotic immediately	T3 Baseline	T4 post-hypnotic long-term
Control	T1 Baseline	Reading	T2	T3 Baseline	T4 (Long-term)

### Procedure and measurements

We tested the effect within the hypnosis group comparing them to their own baseline and between a hypnosis and a control group that did not receive our intervention. Participants in the hypnosis group received a live hypnosis session with suggestions of strength. The original German hypnosis text and an audio recording of it are available via 10.5281/zenodo.10866281. The English translation of the hypnosis text is available as supplemental material. For the hypnosis induction and reorientation part, we followed the SHSS: C (Stanford Hypnotic Susceptibility Scale;^25^). The hypnotists were the first and second authors of this study. Both hypnotists attended hypnosis workshops with Dr. Barbara Schmidt and in the Milton Erickson Gesellschaft für Klinische Hypnose (MEG), a German society for clinical hypnosis where hypnosis experts provide training. They were also supervised by the senior author of the study. During the 40-minute hypnosis session, the feeling of strength was tied to a post-hypnotic anchor, so participants could activate the feeling of strength outside of hypnosis (post-hypnotically—immediately after the intervention and long-term). The control group was reading a book instead of the hypnosis session in the meantime *(‘Total Recall—Die wahre Geschichte meines Lebens’* Arnold Schwarzenegger and Peter Petre, Wilhelm Heyne Verlag München, German version). We measured objective handgrip strength via a hand dynamometer at four time points; subjective strength was measured using a visual analogue scale and a questionnaire at four time points (Table 1).

**Fig. 1:**
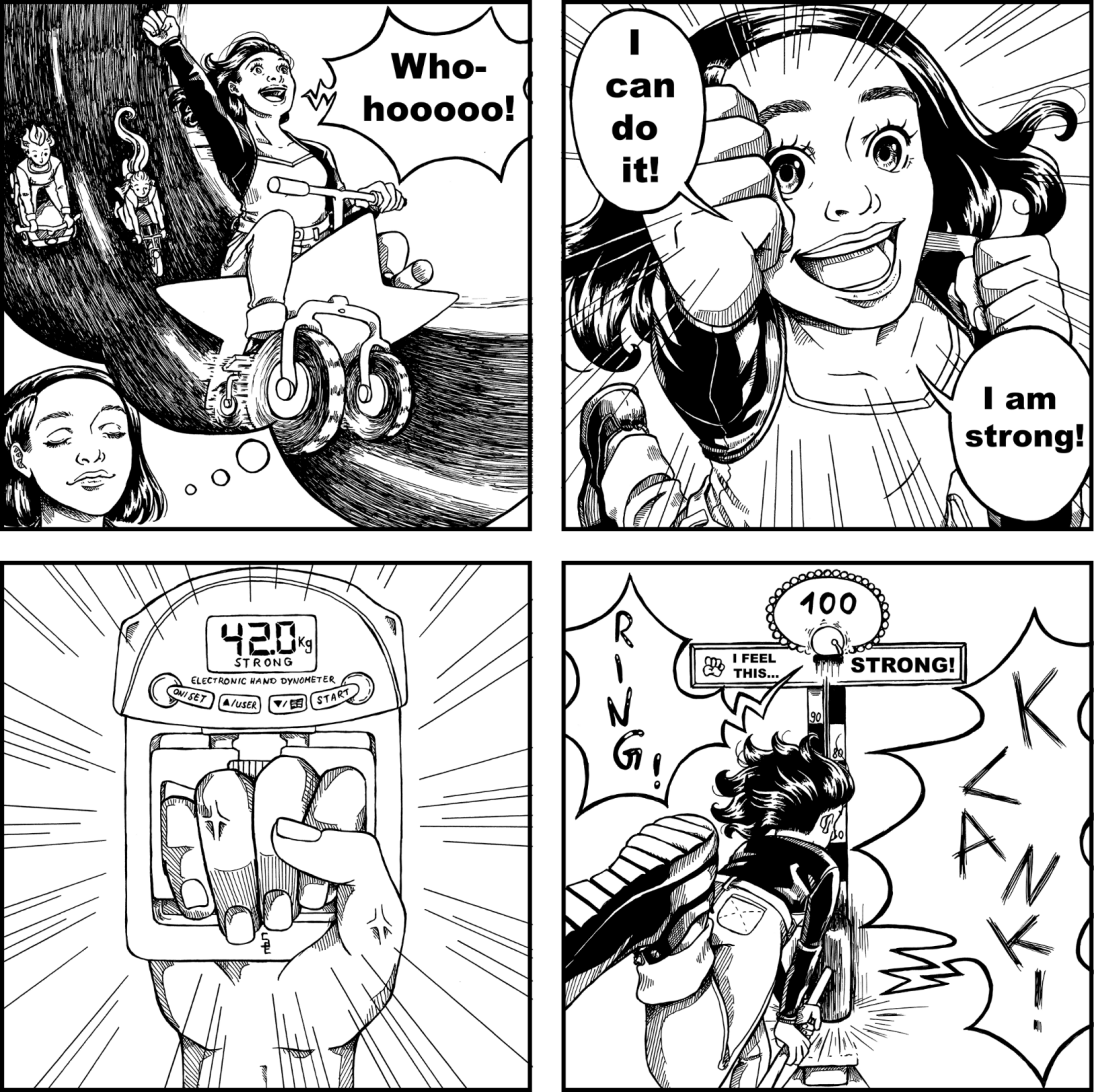
Illustration of our study. (A) Participants in the hypnosis group received suggestions of strength that were linked to a star-shaped power anchor. (B) Participants were instructed to feel strong. (C) Objective strength was measured via hand dynamometer. (D) Subjective strength was measured via a visual analogue scale from 1 to 100. Illustration by Dr. Sophie G. Elschner https://elschner.science/.

We tested both groups on two experimental sessions separated by one week (at the earliest on the 8th day after the first measurement, a few days later was also accepted). To evaluate within-subject effects, baseline measurements were done on both, day 1 and day 8. Table 1 shows the measurement points. At each measurement point both objective and subjective strength was measured. On day 1, baseline handgrip strength was measured (Baseline T1). After the intervention (Hypnosis/Reading), participants of the hypnosis group activated their post-hypnotic anchor. The handgrip strength was measured post-hypnotically immediately (T2). After one week on day 8, baseline measurements were done first (Baseline T3). Participants of the hypnosis group activated their post-hypnotic anchor and measurements took place again (T4 post-hypnotic long-term). Figure 1 illustrates the study design, highlighting the suggested feeling of strength that was tied to a star-shaped power anchor.

### Measuring objective handgrip strength

All measurements using the hand dynamometer were taken in a sitting position with the arm held at the side, the elbow bent to 90°, and the wrist held in a neutral position^26^. At the beginning, participants were demonstrated the correct posture as well as how to use the dynamometer. Participants were also allowed to adjust the dynamometer to fit their hand at the beginning so that they could optimally enclose the handle. Objective handgrip strength is measured in kilograms (kg).

At every measurement point, three strength measurements were done á 5 s with 15 s break. All measurements were taken under the instruction *‘Squeeze as hard as you can now’*.

### Measuring subjective strength

The assessment of the subjective strength was done using a visual analogue scale (VAS). The subjective assessment was made on the statement: ‘I feel strong’. Participants got a 10 cm line where they could place their mark from 0 to 100.

### Measuring hypnotic trance depth

Trance depth was measured using the German ISHD (Inventory Scale of Hypnotic Depth^26^, . This questionnaire comprises 38 items on subjective feelings during hypnosis.

### Measuring the success of the post-hypnotic suggestion

Participants in the hypnosis group were also asked to rate their feeling of strength related to their post-hypnotic power anchor (10.5281/zenodo.10866281). This had to be rated with a number between 1 (no effect) and 5 (highest effect).

### Measuring sportiness

In order to examine correlations between sportiness and physical hand strength as well as the feeling of strength, participants were asked about their sportiness. The number of days on which they played sport and the type of sport were recorded.

### Good scientific practice

This study was performed in accordance with good scientific practice and open science compliance guidelines. All used materials were published online (10.5281/zenodo.10866281).

### Vote on ethics

An ethics application was submitted and approved at the local ethics committee of the Jena University Hospital (No: 2023-3103-BO). All methods were performed in accordance with the relevant guidelines and regulations.

### Randomization and stratification

Participants were randomized and assigned to either the hypnosis or control group by means of an automized tool (paRANDies, Jena University Hospital). Stratification was done by location (Jena and Leipzig) and gender.

### Statistical analyses

Data were analyzed using R Studio (Version R.4.2.3)^28^. All codes were written in English and published online (10.5281/zenodo.10866281).

Significance of the results was assumed for values of *p* < 0.05 (*) with *p* < 0.01 as very significant (**) and *p* < 0.001 as highly significant (***).

Data were analyzed between-groups (hypnosis, control) and within-subjects. Mixed analyses of variances (mixed ANVOA) were done as well as post-hoc *t*-tests. Correlation-tests were done for objective handgrip strength and VAS ratings as well as for the depth of trance and VAS ratings.

For dynamometry measurements, the mean of all three values was used, according to Innes^29^. Published protocols also contain analyses with the highest of all three values (10.5281/zenodo.10866281).

The ISHD was analyzed statistically according to the recommendation of Riegel et al.^27^. The scoring of the depth of trance is categorized as follows: 0–70 low trance depth, 71–94 medium trance depth, 95–136 high trance depth^27^.

## Results

### Sample characteristics

There was no significant age difference between both groups (*p* = 0.078) with a mean of 29.3 years (hypnosis group mean: 26.8, control group mean: 31.9), SD = 10.14. The age range was 18–60 years. Slightly more men took part in the study (hypnosis group: 14 male, 10 female; control group: 14 male, 9 female, 1 gender-queer). Male participants were stronger (50.2 kg) than females (32.5 kg) (*t*_*Welch*_(41.3) = 9.1, *p* < 0.001, d = 2.40) and also felt stronger (*t*_*Welch*_(30.7) = 2.4, *p* = 0.020, d = 0.77) with a mean difference of 11.5. There was no significant correlation between the number of sport days and the objective handgrip strength of a participant (*p* > 0.627) as well as for subjective strength (VAS) (*p* > 0.927). Gender-related statistical analyses were done without the data of the gender-queer participant.

### Objective handgrip strength

A mixed analysis of variance (mixed ANOVA) on objective handgrip strength showed significant effects for the between factor group (hypnosis, control) and the within factor time (T1–T4) for Day 1 (*F*(1, 46) = 6.0, *p* < 0.05) and day 8 (*F*(1, 46) = 13.8, *p* < 0.001). To elaborate these effects further, we conducted within groups and between groups comparisons.

### Within hypnosis group

**Fig. 2:**
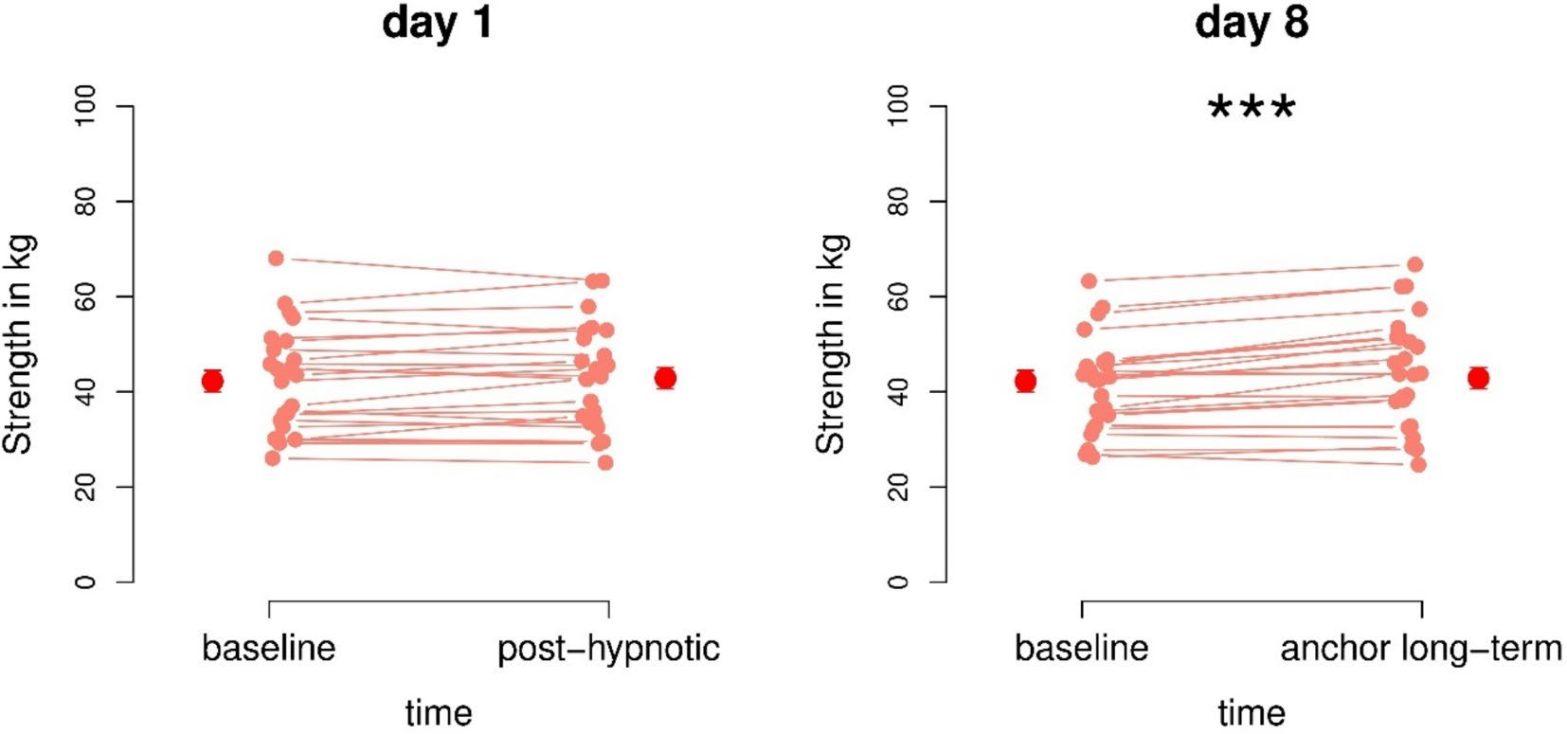
Objective handgrip strength measurements showed highly significant increases after activation of the post-hypnotic power anchor compared to the subject baseline one week after the hypnosis session (day 8). On day 1, we found no significant increase of objective handgrip strength.

In the hypnosis group, data showed no significant differences for objective handgrip strength from baseline T1 to T2 immediately after the hypnosis session (*p* = 0.242), implicating that participants in the hypnosis group were not objectively stronger after the hypnosis intervention. One week later, we found a highly significant increase in objective handgrip strength from baseline T3 to T4 with activated power anchor (*t*(23) = 5.0, *p* < 0.001, d = 1.0) with a mean difference of 2.9 kg, meaning that participants of the hypnosis group were significantly stronger after activating their power anchor compared to the baseline measurement one week after the hypnosis session. Results of the hypnosis group are shown in Fig. 2.

### Within control group

The control group showed a significant decrease of objective handgrip strength from baseline T1 to T2 after reading Arnold Schwarzenegger’s autobiography (*t*(23) = 2.2, *p* = 0.041, d = 0.44) with a mean difference of 1.6 kg. One week after, objective handgrip strength did not differ between T3 and T4 in the control group (*p* = 0.699).

### Between hypnosis and control group

**Fig. 3:**
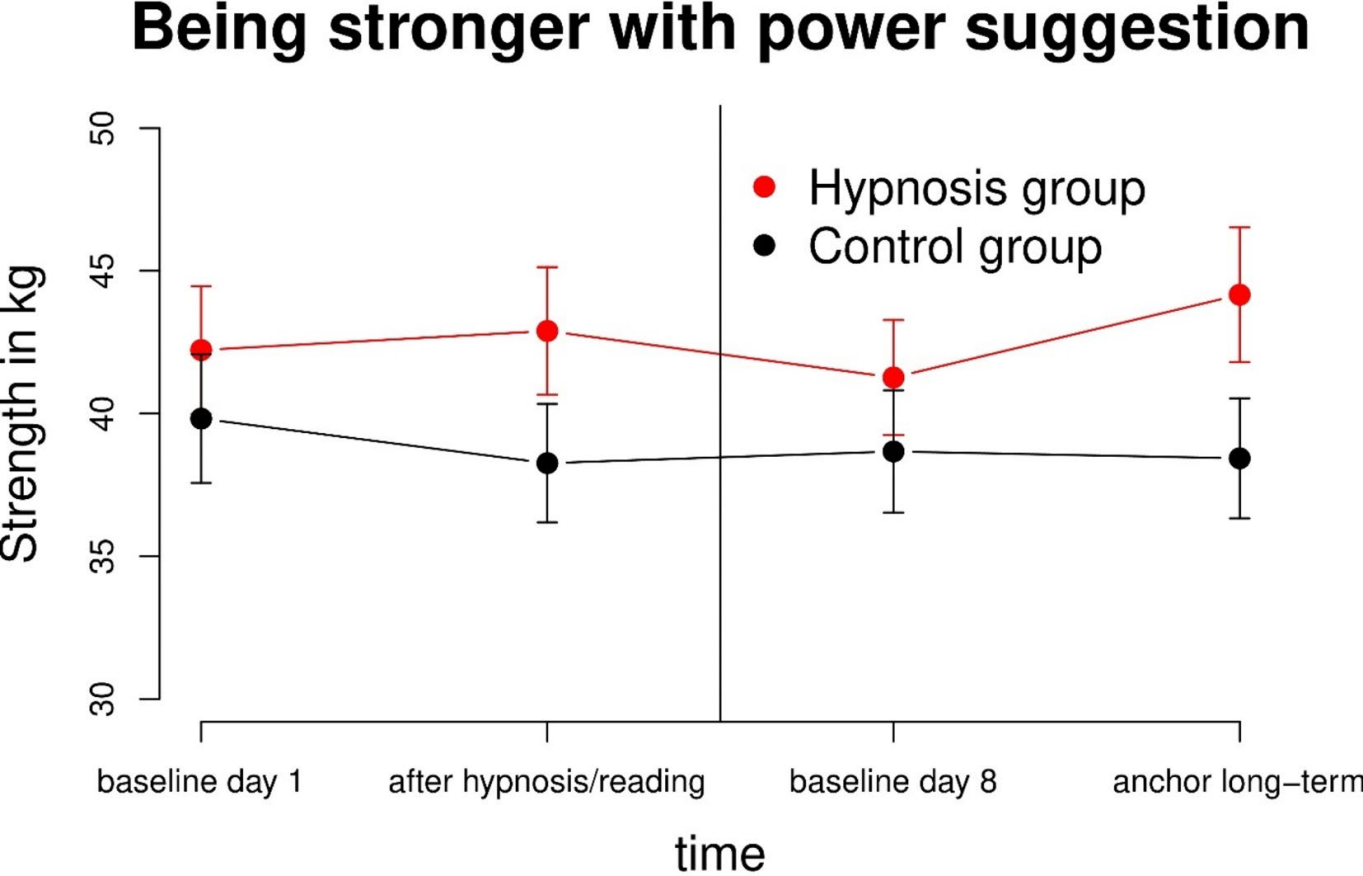
Objective handgrip strength measurements between the hypnosis and control group. No significant differences were found between-groups for all four measurement points, although a tendency was registered. The mean of objective handgrips strength (long-term) T4 (after activating the anchor) within the hypnosis group was 44.2 kg compared to 38.4 kg in the control group.

Objective handgrip strength measurements showed no significant differences between-groups for all measurement points (baseline T1 (*p* = 0.453); T2 (*p* = 0.136); baseline T3 (*p* = 0.383); T4 (*p* = 0.077)) as shown in Fig. 3. Each time we measured objective handgrip strength, we obtained three values. Using maximum values instead of mean values showed similar results (https://doi.org/10.5281/zenodo.10866281).

### Subjective strength

A mixed analysis of variance (mixed ANOVA) on subjective strength showed significant effects for the between factor group (hypnosis, control) and the within factor time (T1–T4) for Day 1 (*F*(1, 46) = 26.5, *p* < 0.001) and long-term (*F*(1, 46) = 14.1, *p* < 0.001). To elaborate these effects further, we conducted within groups and between groups comparisons.

### Within hypnosis group

**Fig. 4:**
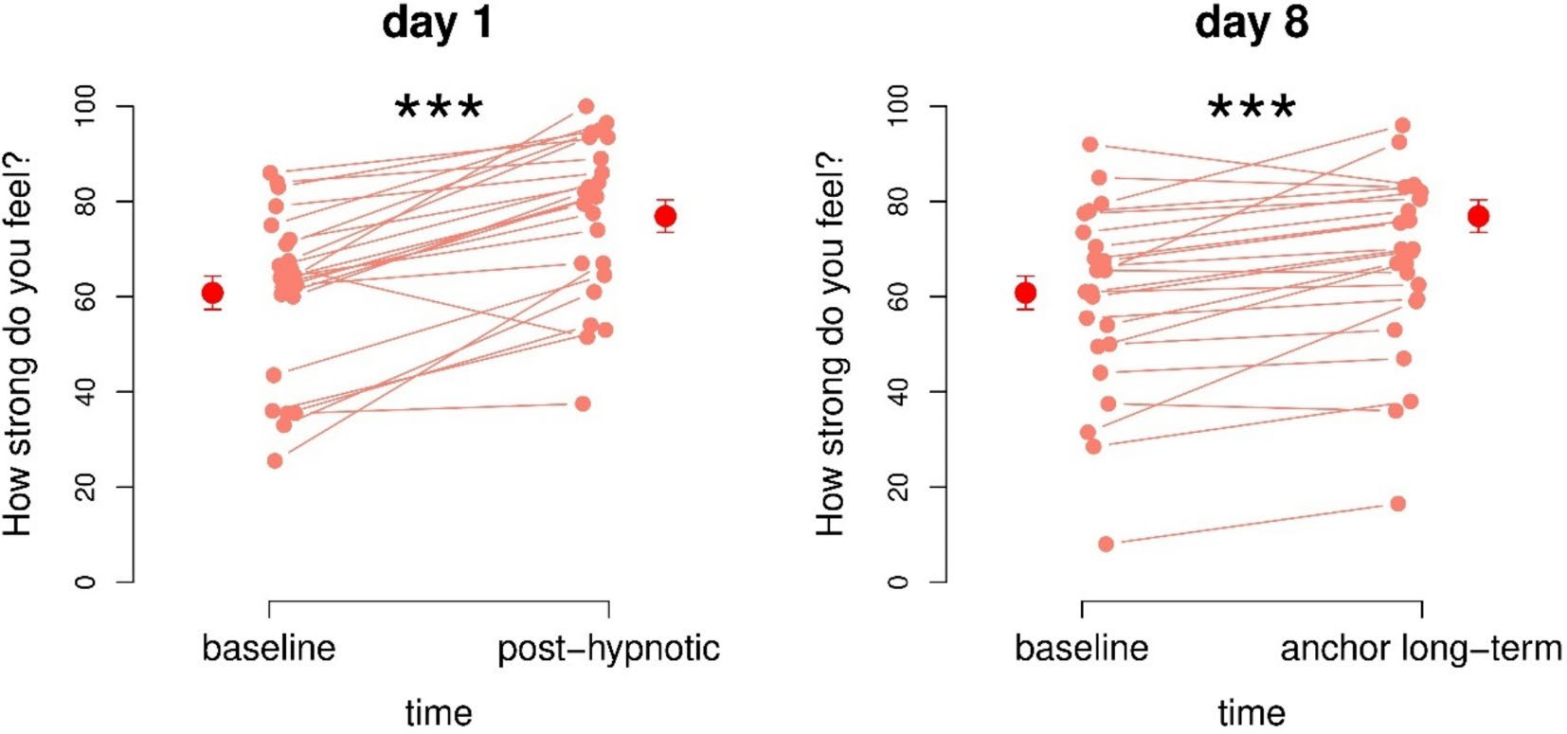
The subjective feeling of strength increased significantly within-subjects on both day 1 compared to subject baseline and on day 8 after activating the power anchor compared to subject baseline.

Participants in the hypnosis group felt significantly stronger from baseline T1 to T2 immediately after hypnosis (*t*(23) = 6.8, *p* < 0.001, d = 1.39) with a difference in mean of 16.1 (Fig. 4, left side). The feeling of strength also increased significantly within-subjects from baseline T3 to T4 one week after the hypnosis session after activating the power-anchor (*t*(23) = 4.4, *p* < 0.001, d = 0.89) with a difference in mean of 7.5 (Fig. 4, right side).

The effects of the post-hypnotic power suggestions were rated from 1 to 5 (success of post-hypnotic suggestion). The feeling of strength related to the anchor during hypnosis (success of post-hypnotic suggestions T1) was rated with a mean of 3.67 and was highly significant (*t*(23) = 13.0, *p* < 0.001) compared to no effect with a large effect size of Cohen’s *d* = 2.6. After the hypnosis intervention, the success of post-hypnotic suggestions T2 was rated with a mean of 4 and also showed highly significant results (*t*(23) = 22.3, *p* < 0.001, *d* = 4.5). The mean of the long-term scoring on day 8 (success of post-hypnotic suggestions T3) was 3.33 with highly-significant results (*t*(23) = 14.0, *p* < 0.001, *d* = 2.9).

#### Between hypnosis and control group

**Fig. 5:**
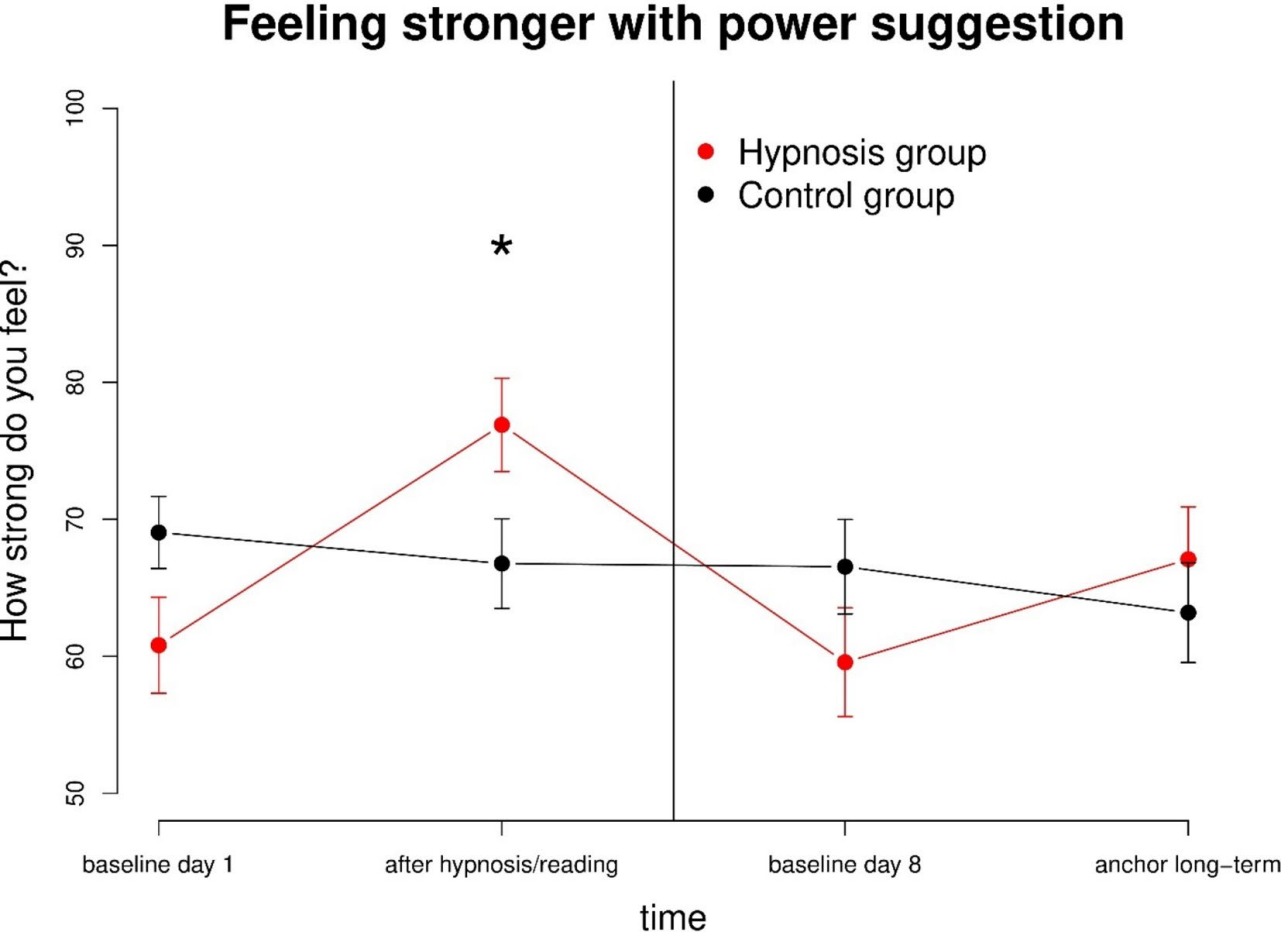
Subjective strength was significantly increased in the hypnosis group after the intervention compared to the control group. Participants in the hypnosis group did not feel significantly stronger on day 8 after the activation of the anchor compared to the control group.

Measurements of subjective strength were equal in the beginning of the study at T1 in both groups (*p* = 0.067), although the control group tended to feel stronger (mean hypnosis group 60.8, mean control group: 69.0). After the intervention (hypnosis/reading) at T2, participants in the hypnosis group (mean 76.9) felt significantly stronger (*t*_*Welch*_(45.9) = 2.1, *p* = 0.037) than in the control group (mean 66.8) with a medium effect size (Cohen’s *d* = 0.62) (Fig. 5). We also found no difference between group baselines one week later (VAS baseline T3, *p* = 0.191) with a mean of 59.6 for the hypnosis group and a mean of 66.5 for the control group. No significant differences were found for long-term effects between-groups (VAS (post-hypnotic long-term) T4, *p* = 0.465) with a mean in the hypnosis group of 67.1, and a mean in the control group of 63.2 (Fig. 5).

### Correlation of objective and subjective strength

Correlations were found between objective and subjective strength. Higher objective handgrip strength was significantly associated with higher subjective handgrip strength at baseline T1 (*r* = 0.32, *p* = 0.028) as well as T2 (*r* = 0.31, *p* = 0.034). No significant correlations were found for T3 (*p* = 0.107) and T4 (*p* = 0.059), indicating that the subjective assessment did not match the objectively measured strength on day 8.

### Depth of trance

Participants in the hypnosis group experienced a medium trance depth (mean = 93, SD = 13.3). We found no significant correlation for the increase in subjective strength (Delta VAS – Difference from T2 to Baseline T1 and T4 to Baseline T3) and the depth of trance from T2 to T1 (*p* = 0.065) and T4 to T3 (*p* = 0.504). Depth of trance did also not correlate significantly with the suggestion success immediately after the hypnosis intervention (*p* = 0.732) and post-hypnotic long-term (*p* = 0.263).

To determine the item intercorrelation of the ISHD, the internal consistency was calculated and resulted in excellent values (Cronbach’s alpha = 0.9).

### Differences between hypnotists

As an exploratory analysis, we added the between-subjects factor place (Jena, Leipzig) in our ANOVAs as we had two different hypnotists on both sites. We found no significant main effects of place, so we conclude that both hypnotists achieved similar effects. The only significant interaction emerged on the second experimental session and shows that participants in the hypnosis group measured in Leipzig showed a highly significant increase of objective handgrip strength compared to Jena.

## Discussion

In our study, we show that the suggestion of strength given during hypnosis increased both objective and subjective strength parameters. Subjective strength increased significantly when participants activated their post-hypnotic power anchor compared to their own baseline, both immediately after the hypnosis session and one week later. We also found significant effects between the hypnosis and the control group immediately after the hypnosis session. The hypnosis group felt significantly stronger after activating their power anchor compared to the control group that was reading Arnold Schwarzenegger’s biography instead of hypnosis. Our data therefore indicate that our hypnosis intervention significantly improved strength.

Due to the feedback of our participants, our hypnosis intervention made them feel stronger and more self-confident. One week after the hypnosis session, some participants said that they used the post-hypnotic anchor while climbing and felt trust in their own skills and confidence. One participant also told us that she would feel like Hulk when activating the power anchor. This feedback supports our results of changes in participants’ perception.

Objective strength significantly increased 1 week after the initial hypnosis session when participants activated their post-hypnotic power anchor compared to their own baseline, but not immediately after the hypnosis session. This might be due to the relaxing effect of hypnosis that interfered with squeezing the hand dynamometer. Some participants felt still very relaxed after the hypnosis session (carry-over-effect of relaxation) and still had to process the experience, even though they were already fully awake again. The significant decrease in objective handgrip strength in the control group after reading Arnold Schwarzenegger’s autobiography compared to their own baseline indicates that reading did not motivate participants the same way our hypnosis intervention did.

Our data do not show a discrepancy between objectively measured strength and perceived subjective strength, but subjective strength was the parameter that changed first and most prominent. Based on these results, we suggest that our hypnosis intervention primarily affects the mental state of participants and that the subsequent physical parameter changes are a consequence. This is in line with Schmidt et al.^30,31^ who showed that when you block participants’ vision or hearing during hypnosis, primary perceptual processes are not affected, but subsequent cognitive processes are. An altered mental state might lead to altered physical parameters via altered information flow in the brain^31,32^. It would also confirm the literature that a voluntary muscle reserve can be activated through hypnosis^33^. Increased objective handgrip strength might be due to a higher recruitment of motor units by a changed neural control within the muscles^34,35^.

Conducting the study in two different places by two different people required a high standardization of procedures. We used a SOP (Standard Operating Procedure) that defines the processes, the procedure and the verbal instructions. We found no significant main effects of the two places/hypnotists on our experimental effects which indicates the generalizability of our findings.

There are some limitations to this study. We did not test participants’ hypnotic suggestibility before the main experiment because Schmidt et al.^18^ showed that hypnotic suggestibility does not affect the efficacy of hypnotic suggestions and is therefore not necessarily a predictor for the outcome of a hypnosis intervention^36^. Instead, we measured hypnotic depth that significantly correlates with participants’ hypnotic suggestibility^27^. Most of our participants reached a medium to high trance depth, so we can assume that their hypnotic suggestibility was also medium to high.

The literature states that very experienced participants who may have already reached their physiological strength limits would not be able to improve their performance through hypnosis (ceiling effects;^37^). We did not exclude trained participants; on the contrary, several people interested in climbing and bouldering participated in our study, because they wanted to improve their handgrip strength. The sportiness of our participants had no influence on the results. Therefore, we assume that our participants had not yet reached their physiological strength limit.

Many potential influencing factors were not excluded, e.g. training between the two appointments or the use or non-use of the post-hypnotic power anchor during the week between the two experimental session. Another influencing factor might have been habituation with the hand dynamometer as well as acclimatization to the situation or the general well-being of the participants. Participants may also have deliberately influenced their strength in order to meet the expectations of the experimenters. Furthermore, we ran the study during winter, so participants arrived with cold hands which could have affected how they squeezed the hand dynamometer.

Our study design excluded the possibility of doing a double blind study. Both the participants and the experimenters knew which group (hypnosis, control) the participants belonged to. Therefore, we cannot exclude a possible influence on subjective evaluations due to altered expectations on both sides.

Although we used suggestions relating to already existing data^22^, it might be useful to add suggestions of physiological facts, e.g. that there is a 30% reserve within voluntary muscles that can be activated through hypnosis^33^.

With our study, we show that a single hypnosis session improves subjective and objective strength parameters. We also show that participants could re-activate their feeling of strength via a post-hypnotic power anchor that worked for at least one week. With our study, we contribute interesting results to the current state of research, emphasizing the power of mind to influence both perception and physical parameters. Our study also indicates practical relevance. It shows that hypnosis is an effective, non-invasive method to increase strength, both subjectively and objectively, for professional athletes to improve their performance and for patients to improve their medical condition.

### Implications for practice and future research

Further research might focus on different groups and investigate the duration of the long-term effects of the hypnosis intervention beyond one week after the hypnosis session. Studies could also include a third group of participants receiving strength suggestions without hypnosis. Previous studies already showed the influence of hypnotic suggestions on physiological parameters^12,38,39^. Further research might focus on the detailed neuronal mechanisms that lead to these alterations and might be able to explain the chain from mental alterations over neuronal mechanisms to changed behavior or changed physical parameters. Understanding the effects of hypnosis on muscle strength can contribute to scientific knowledge and may uncover the effects of the impact of the mind on physical functions to further understand their complex interplay. It might help to understand psychological factors that affect the body and that can be used for therapeutic purposes.

Our hypnosis intervention can help patients to improve their medical condition. As a non-invasive therapy, it might help patients after a long disease or a heavy surgery to regain their strength. Patients often suffer from weak bodies^40–42^ and bad mental states (e.g. depression^43,44^), . Clinicians repeatedly report that the patient’s mental state and inner feelings are essential influencing factors for recovery and outcome^45–48^. The feeling of the own ability to reach these goals is crucial. Our intervention offers an excellent opportunity for this. Also, patients who suffer from chronic fatigue and/or post-exertional malaise such as ME/CFS-patients (myalgic encephalomyelitis/chronic fatigue syndrome and also long COVID- and post-vac-patients) might profit from our intervention. ME/CFS is a neuro-immunological disease with a complex clinical profile. Post-exertional malaise (PEM), a pronounced and persistent intensification of all symptoms after minor physical or mental exertion, is typical of ME/CFS. Patients often experience their bodies as weak^49^ and lose confidence in the body^50^. ME/CFS is often associated with anxiety, which can impair fatigue symptoms^51^. In addition, patients with ME/CFS in particular are still often marginalized as psychosomatic^52^ and often do not receive adequate treatment^53^. Interestingly, in ME/CFS patients, fatigue intensity seems to correlate with handgrip strength and cytokine level^52^. Our intervention might help to become physically more stable again. Studies could already show that hypnosis can help improve fatigue symptoms in chronic fatigue patients (self-hypnosis and neurofeedback^54^).

Also, professional athletes could profit by using our intervention. Athletes try to push their limits more and more. This has led to athletes overtraining and breaking down^55^. The increase in performance according to the motto ‘more is more’ has reached its limits. Cognitive strategies to improve performance are already used in professional sports. Athletes usually have an individual mental state of maximum performance^56^. Suggestions and key words are already used to achieve this^57^ or remember a positive experience of success^58^. Tod et al.^59^ mention that these strategies probably alter the central-nervous system [see also^60^], leading to altered motor unit recruitment, synchronization and firing rates within neurons: implying that changes in the central nervous system could possibly influence the activity of the sympathetic nervous system. This could lead to changes in peripheral elements, such as the ability of muscles to contract. These alterations in muscle function might manifest in the main muscles driving the movement, the opposing muscles, or any other muscles involved in the motion. Our intervention represents an opportunity to use inner resources by combining these approaches and using the focused attention and heightened sensory and imaginative understanding within a hypnotic trance state in a way that has not yet been harnessed.

## Conclusion

In our study, we show that one hypnosis session where the feeling of strength is tied to a post-hypnotic power anchor can increase subjective and objective strength with effects lasting at least over a week. Our findings might be of relevance for medical and sports practice. Our intervention suggests the possibility for a cost-effective, non-invasive and multi-level approach that might help patients regain their health and athletes improve their sports performance. We suggest using the power of mind as the mental state influences a person’s health and performance outcomes. We emphasize the importance of considering both psychological and physiological effects. Hypnotic suggestions might lead to alterations in the brain and probably act through changes in the mental state, which causes effects on the physiological level.

## Electronic supplementary material

Below is the link to the electronic supplementary material.

Supplementary Material 1

## Data Availability

After the positive vote on ethics and before recruitment of participants, the study was preregistered with the German Registry of Clinical Studies (Deutsches Register Klinischer Studien, DRKS; DRKS00032788; https://drks.de/search/de/trial/DRKS00032788).Statistical protocols were also published online (10.5281/zenodo.10866281).All analogue data are stored at Jena University Hospital (Institute for Psychosocial Medicine, Psychotherapy and Psychooncology). Used materials are also published online according to open science guidelines (cf. Good scientific practice, 10.5281/zenodo.10866281).
