# Supplementary material for: Increasing handgrip strength via post-hypnotic suggestions with lasting effects
Source: Sci Rep. 2024 Oct 14;14:23344. doi: 10.1038/s41598-024-73117-0 (PMC11473724; doi:10.1038/s41598-024-73117-0)
Supplement: Supplementary file 1 — Supplementary Material 1 [file 41598_2024_73117_MOESM1_ESM.pdf]

Hypnosis with strength suggestion

Results reported in:

Nieft, U., Schlütz, M., & Schmidt, B. (2024). Increasing handgrip strength via post-hypnotic suggestions with lasting effects. *Scientific Reports*

Hypnosis introduction (SHSS:C; Weitzenhoffer & Hilgard, 1962)

Let's start our hypnosis session now. I want you to relax as much as possible and be able to sit comfortably in the chair.

Today we want to examine how you will react to hypnotic suggestions. Of course, nothing will happen here against your own will or that could expose you in any way.

Just pay attention to what happens and try to allow the experiences that I will suggest to you. Don't push yourself to do anything that doesn't work, but try not to hold back the things that do work. You can do absolutely nothing wrong. Just be completely relaxed.

Do you see this sign in front of you? (Show a fixation point in front of the participant)

Very good. Just relax in the chair, focus your gaze on the sign and listen to my voice. In the meantime, I will give you some instructions that will help you to relax and gradually enter a hypnotic state.

Please keep your eyes fixed on the sign and continue to pay attention to my words as you look at it. You can only enter a hypnotic state if you want to. Try your best to concentrate on the sign and pay close attention to my words. If your mind wanders, simply bring your thoughts back to the sign and my words. Pay attention to how the sign changes, how it perhaps sometimes becomes unclear, sometimes clearer. Whatever goes through your mind, allow it to, but keep focusing on the sign.

Relaxation in hypnosis is very similar to the state just before falling asleep, but you will not sleep in the usual sense because you will continue to hear my voice and be able to focus your thoughts on what I tell you.

You are pleasantly relaxed, but you will relax even more, much more. Just pay attention to my voice. Sometimes my voice may seem to change or sound like it's far away. That's okay. If you start to feel sleepier, that's okay too. Accept whatever happens and just keep listening to my voice as you relax more and more.

As you visualize the relaxation, your muscles will relax. Start with your right foot, relax the muscles of your right leg... now the muscles of your left leg... relax completely. Relax your right hand, your forearm, your upper arm and your shoulder... now your left hand... and your forearm... and upper arm... and shoulder... relax your neck and your chest... completely relaxed.

As you relax more and more, your body will feel heavy or perhaps numb. You will start to feel this numbness or heaviness in your legs and feet... In your hands and arms... In your whole body... As if you are sinking deeper and deeper into the chair. The chair is stable, it will hold your body as it feels heavier and heavier.

Your eyelids feel heavy, very heavy. You start to feel relaxed and sleepy. Your eyes burn a little and your eyelids feel very heavy. Your eyelids are getting heavier...

Your eyes become blurred from the strain. You can barely see the sign; your eyes are so strained. Soon you won't be able to keep your eyes open. Your eyelids are heavy. Very heavy. Getting heavier and heavier. They press down, lower and lower. There seem to be weights on your eyelids, pushing them lower and lower.... Your eyes flicker... flicker... close, close.

Your eyes are now closed. Just keep your eyes closed until I ask you to open them.

You are relaxed, very relaxed. You can relax even more if you simply let yourself go. You can reach a state of even deeper, complete relaxation. You become increasingly sleepy. You feel a pleasant sensation of numbness and heaviness throughout your body. You begin to feel so relaxed, so sleepy. It's easier to turn your thoughts away from other things and focus your attention solely on my voice. Soon you will just sleepily listen to my voice as you relax more and more.

Now I want you to pay attention to your breathing ... Take a deep breath ... a deep breath. Then hold your breath ... And then breathe out through your mouth all at

once. You may already feel calm and relaxation spreading through your body ... Now breathe in and out calmly and evenly again - in and out ... As you inhale, your abdominal wall arches slightly upwards, and as you exhale it falls slightly downwards again ... in ... and out ... in ... and out. How this inhaling and exhaling goes hand in hand with calmness and relaxation! If you continue to breathe calmly this way, sooner or later you will reach a very pleasant inner peace without having to do anything to achieve it... You breathe in and out ... in and out. Whenever you breathe in, you take in oxygen and energy ... And every time you breathe out, you relax more deeply and can let go ... With every breath you take, you can let go more ... with every single breath ... You breathe out and let go of everything disturbing ... You breathe out and inner peace will come more and more - all by itself ... And with every breath, the relaxation becomes deeper and deeper ... as deep as you can now.

All you need to do is pay attention to your breathing and feel how your breath flows in and out ... in and out ... And you can imagine how your breath flows through your nose via your windpipe to your lungs and back again...

Some people focus their attention on the muscles of the body and feel how they relax deeper and deeper with each breath, as if the breathing is telling the muscles to relax even further ... You don't need to do anything ... Just feel and notice. You can often feel the relaxation first in the temple area. It moves across the forehead to the eyes ... to the root of the nose over the cheeks and the nostrils ... over the lips into the mouth.

And this relaxation also spreads to the neck, because with every breath the muscles there also become more relaxed and looser ... You don't have to do anything. Simply feel and notice how the relaxation flows away - like a stream - over your shoulders, down your back ... Every time you breathe out, you can feel the relaxation - even in your upper arms ... in your forearms ... and finally your fingers join in, the palm of your hand ... right down to your fingertips ... And as you breathe out, the air flows gently and smoothly back out through your nose ... This allows your arms to relax - all by themselves ... With every breath, calm and relaxation become more and more ... If you pay attention to your nose, you can feel how the air flows in and out through

your nose ... And you can feel how the whole head area, the upper body and your arms and hands are gradually gripped by a deep feeling of relaxation and calm ... As you inhale, you may have the sensation of becoming lighter ... This is quite normal... You may have noticed that the exhalation takes a little longer than the inhalation... So with each breath you sink deeper ... and deeper ... and deeper.
You are relaxed, very relaxed. Your whole body feels heavy and relaxed. You feel a pleasantly warm sensation throughout your body as you become more and more sleepy. Sleepy. Very sleepy. Keep focusing your thoughts on what I am saying; listen to my voice. Soon you will think of nothing but my voice and the words I am saying as you relax more and more. There is nothing you need to worry about now. Nothing but what my voice tells you seems important, nothing else is important now. Even my voice may sound a little strange, as if it comes from a dream, as you sink deeper into this numbness, this heaviness of deep relaxation. Relax completely...

I will soon start counting from 1 to 10. As I count, you will notice how you fall deeper and deeper into a restful sleep. But you will still be able to do all the things I ask you to do without waking up...

1... You begin to relax even deeper... 2 .... Deep, deep into a deep healthy sleep... 3, 4... deeper and deeper sleep... 5, 6, 7, ... You sink into a deep, deep sleep. Nothing will disturb you... Please direct your thoughts to my voice and to the things I tell you. You will experience many of the things I describe to you... 8, 9, 10... Deeply asleep. You will not wake up until I ask you to.
I want you to realize now that you will be able to speak, move and even open your eyes when I ask you to, and remain in the hypnotic state you are in now. Whatever you do, you will remain in the hypnotic state until I tell you otherwise...

Hypnosis test (1st item of the SHSS:C; Weitzenhoffer & Hilgard, 1962)

Now stretch your right arm out at shoulder height with your palm facing upwards. Just like this. Pay close attention to your right hand, how it feels, what is going on in

it. Notice whether it feels a little numb or not, or perhaps tingling. Now imagine that you are holding something heavy in your hand, perhaps a heavy ball or a billiard ball... something heavy. Form your fingers around it as if you were holding this heavy object you are imagining in your hand.

Now your hand and arm feel heavy, as if a weight is pushing them down... your hand and arm start to move down... as if they are being pushed down... they sink... sink... lower and lower... The arm becomes more tired and strained... lower... slowly but surely... down, down... The weight is so great, the hand is so heavy... You feel the weight more and more... The arm is too heavy to hold back... It moves down, down... lower and lower...

Good. Simply return your hand to its original resting position and relax. Whatever you do, you will remain in the hypnotic state until I tell you otherwise.

Strength suggestion (Nieft, Schlütz & Schmidt, 2024)

I would like to go on a nice journey with you now. We're going to a place together where you feel very comfortable and safe. A place that you may know. Perhaps a place that you don't yet know and that is just emerging in your mind's eye. This place is very familiar to you in a very pleasant way. Feel inside yourself, what impressions does your safe, beautiful place awaken in you? What do you see? What do you feel? What do you smell in your place, which you can now decorate in your innermost being exactly as it feels most harmonious to you... and where you feel completely safe, comfortable and cared for. In this place, you are very close to yourself ... and very close to your sources of strength, to your very own energy, to the source of yourself ... While you are in your wonderful place and enjoying the pleasant feelings - you can relax and listen to my voice and what it is saying to you.

I would now like to help you activate your inner source of strength. The fact is that every person, including you, has their own treasure chest. This treasure chest is filled

to the brim with treasures and surprises. Imagine your own treasure chest. What exactly does it look like? And imagine opening it and looking inside. Perhaps you will see sparkling and shining diamonds and valuable objects. This treasure chest contains all the skills you have and could ever need. Most people underestimate their own abilities. They are not aware of their precious treasure, it lies dormant in secret. And it is the same with physical performance. Most people don't realize that they can do and achieve much more than they previously believed.

Earlier you squeezed the grip strength meter and thought you were squeezing with full force. At a certain point you had the impression that you couldn't squeeze any harder, that you had now given it your all. This happens because we interpret feelings of exertion and fatigue as a signal that we are at our limit. But that's not true. I will now tell you how you can make use of your inner treasure. First and foremost is always your will, your decision to make use of your inner treasure - your will to give everything you have to give. It is also important that you realize that feelings of effort, discomfort and fatigue are signs that you can go further and that your true limit is a little further beyond. And how good it feels to find out that there is so much more!
So that you can utilize all of this, I would now like to work with you to find access to the source of your strength. To do this, think of a situation in which you felt really strong and powerful. Perhaps you overcame a major challenge and you are happy and proud of what you have achieved. Go back to this situation again. How exactly did you feel? Was it perhaps a warm and strong feeling in your chest? Can you feel it again right now? This chest swelling with pride, this feeling of being able to tear down trees? What is it like to feel so strong? Perhaps you have also seen athletes after a big physical challenge. What did it look like when they were so happy about their own performance, beaming with joy and pride? Perhaps the way you felt and the way you feel now. Feel inside yourself. In which part of your body do you notice this feeling of strength most clearly?

When you have found this place in your body, concentrate on it and thereby increase the feeling. The feeling of strength grows, just like a plant that gets plenty of light and water and stands on nourishing soil. The good feeling of strength spreads further throughout your body and makes you feel capable of anything. You also feel a deep sense of confidence. Confidence in your own strength. Allow yourself to feel so strong. Once again, think about the place in your body where you feel your strength. From there it radiates out into all parts of your body like rays of sunshine. And you know how strongly the sun can radiate. It is a thoroughly pleasant feeling. You are completely filled with it. The feeling gets stronger and stronger. It becomes so great that it can even be felt outside your body like a shell. The feeling of strength is like a radiance that shines from your body and that others can see and feel. You literally radiate this feeling of strength. I'm about to start counting from 1 to 10 and then you'll feel completely strong. With every number, your feeling of power and strength doubles and you go deeper into trance.

1 ... you feel stronger and stronger... 2... You sink into twice as deep a relaxation and feel even stronger 3... 4 ...and you sink even deeper 5... 6... You now feel very strong 7... 8... 9... 10...

You can break all your records. You remember your inner treasure. Now you know how to use it. And you let any inhibitions that could hinder your performance diminish until they disappear completely. Any sign of discomfort or tiredness while you are pressing the grip strength meter will be an incentive to keep going. You will realize that you can do more than you thought! And that's a wonderful feeling! Just concentrate on pressing the meter and you'll be able to perform at your absolute best. There is suddenly much more concentration, effort and willpower. It feels really easy and you just feel like giving it everything you've got. Think about how you will feel the effort in your whole body. How alive you will feel. And that you will use all your strength to push even harder.

Post-hypnotic power anchor (Nieft, Schlütz & Schmidt, 2024)

There's a very simple thing you can do to recall this feeling of strength. Perhaps you know the game Super Mario Kart, where you race a with a character. Remember what it was like to play this game. You drive over one of those yellow question mark boxes and then the star appears in your box at the top. And you know what this star means. When you activate the star, you are super-fast and strong. You are invincible. You glow in all rainbow colors. There is a special music you hear, a melody that accompanies the star and shows you that you are now unleashing your superpower. You can also imagine another star, one from your imagination, for example. One that you like, that sparkles and shines. With this star, you are invincible and can overcome all challenges with ease. Or you can also think of your treasure chest, which is filled with all your abilities. You can use it to recall that feeling of strength again. Suddenly you can do things that you didn't think you could do. How free and easy it feels! You can achieve so much!

Now I would like you to imagine something so that you can reactivate the star or the abilities from your treasure chest. Imagine that you stretch out your hand in front of you and spread all your fingers apart. Look at your hand in your imagination. It looks like a star! And this is how you can activate your star. If you think of this movement and visualize your hand in your mind's eye with your fingers outstretched as if it were a star, you can feel your great strength again, how you can easily cope with anything. You can recall the feeling of strength again and let it flow into a specific part of your body, for example your hand and arm.

Memorize this exactly. Stretch your fingers so that your hand looks like a star. This will automatically give you a feeling of strength. Go through this process again in your mind. You stretch out your fingers and see your star hand. When you do this later, you will naturally and automatically experience this feeling of strength and invincibility again. You enjoy the fact that you now have the opportunity to simply have this good feeling again. Knowing that you can recall that feeling of strength

again at any time, in any place and in any situation feels very good. You visualize your hand in your mind's eye, stretch your fingers, see the star and feel completely strong and confident again. And whenever you use this, you will perform better than ever before. You will be amazed to discover the true extent of your abilities. You will realize that you feel stronger, more stable and more capable and that you can give your absolute best when you recall your strength again.

You are already looking forward to the moment when you recall the feeling of strength again by stretching out your fingers and activating your star.

Reorientation after hypnosis (SHSS:C; Weitzenhoffer & Hilgard, 1962)

Now imagine how you return to the here and now.

If I count backwards from 10 right now, you'll slowly come back. When I get to 1, open your eyes and you will be fully present again. You will also feel how your relaxation turns into a pleasant feeling of being rested and you will end up feeling fresh and alert. Right, I'll start counting.

10 ... 9 ... 8 ... 7 ... 6 ... 5 ... 4 ... 3 ... 2 ... 1

You've returned to the here and now. That's great. Stay relaxed and listen to me. Enjoy these moments a little longer. Your breathing is accompanied by a feeling of freshness in your lungs and you feel increasingly relaxed. I would now like you to consciously take a few more breaths and inhale deeply through your nose and exhale audibly through your mouth. Feel how you become livelier and fresher with each breath, how confidence and new energy rise within you. When you are on the last one of these breaths, you can tense your muscles and stretch out and are completely back in the here and now.
